# Ocular Surface Disorders in a Group of Egyptian Children with End Stage Renal Failure on Dialysis: A Cross-Sectional Study

**DOI:** 10.1155/2016/4767819

**Published:** 2016-01-28

**Authors:** Mohamed Anbar, Ahmed R. H. Ahmed, Abdel-Azeem M. El-Mazary, Ramadan A. Mahmoud

**Affiliations:** ^1^Department of Ophthalmology, Faculty of Medicine, Sohag University, Sohag 82524, Egypt; ^2^Department of Pathology, Faculty of Medicine, Sohag University, Sohag 82524, Egypt; ^3^Department of Paediatrics, Faculty of Medicine, Minia University, Minia 61519, Egypt; ^4^Department of Paediatrics, Faculty of Medicine, Sohag University, Sohag 82524, Egypt

## Abstract

*Purpose*. To investigate tear function, ocular manifestations, and squamous metaplasia of the conjunctival epithelium (SMCE) in children with end stage renal failure (ESRF) on dialysis.* Methods*. Thirty children with ESRF and 20 age and sex matched controls during the period from January 2014 to May 2015 underwent full ophthalmologic examination and the tear function was investigated by the Schirmer and tear film break-up time (TBUT) tests. SMCE was evaluated by impression cytology and immunocytochemistry. The correlations of tear function status with ESRF-related clinical and biochemical variables were measured statistically.* Results*. Dry eye symptoms were detected in 26% of children with ESRF, compared with none of the controls (*P* = 0.05) and SMCE was almost absent. Values of the Schirmer and TBUT tests were significantly lower in children with ESRF for right eye (*t* = 24.63, *P* = 0.01, and *t* = 11.9, *P* = 0.002, resp.) and left eye (*t* = 24.7, *P* = 0.02, and *t* = 11.4, *P* = 0.0004, resp.). TBUT and the Schirmer test values were correlated inversely with the duration of ESRF (*R* = −0.45, *P* = 0.01, and *R* = −0.46, *P* = 0.01, resp.) and with the duration of dialysis (*R* = −0.39, *P* = 0.03, and *R* = −0.45, *P* = 0.01, resp.). None of the following parameters was associated with distorted tear function including serum creatinine, electrolytes, parathyroid hormone, total protein, albumin, CBC parameters, and systolic or diastolic blood pressure.* Conclusion*. The basal tear secretion and tear film stability were lower while the dry eye symptoms such as itching and redness were more common among children with ESRF. The duration of ESRF and dialysis duration seem to be related to the disturbances in tear secretion and tear film stability. However, SMCE is very rare.

## 1. Introduction

End stage renal failure (ESRF) affects the function of many organs including the eye. Ocular complications are frequent in adult patients with ESRF treated with maintenance hemodialysis. For example, retinopathy, retinal detachment, cataract, redness, and irritated eyes may be associated with elevations in the calcium-phosphate product. In those patients, squamous metaplasia of the conjunctival epithelium and corneoconjunctival calcification may occur [1, 2]. Other eye conditions include macular oedema, ischemic optic neuropathy, elevated intraocular pressure, retinal detachment, and retinal haemorrhage may occur less frequently [3].

Studies that evaluated tear function among children with ESRF treated by maintenance dialysis, through midline research, are generally deficient. One previous report showed that dry eye manifestations are frequent in chronic dialysis patients and the tear function statuses are affected on prolonged duration of dialysis [4], Behbehani et al. [5] demonstrated the prevalence of ocular manifestation in children with ESRF before renal transplantation, and Krause et al. [6] found retrospectively some ocular complications in postrenal transplantation children.

However, ocular surface changes, mainly squamous metaplasia of the conjunctival epithelium, may develop in patients with ESRF undergoing hemodialysis in adult [7–9]. To our knowledge there are no previous investigations for the status of the conjunctival epithelium in children with ESRF. Therefore, in this study, the amount of tear production, the stability of the tear film, and the status of conjunctival epithelium in children with ESRF on maintenance dialysis were investigated and correlated with different clinical and laboratory parameters.

## 2. Materials and Methods

This is a prospective case-control study performed on 30 patients with ESRF on regular dialysis at the dialysis unit of Paediatric Department, Sohag University, Egypt, during the period from January 2014 to May 2015. Children underwent dialysis three times weekly by Fresenius 4008S machine (Fresenius Medical Care AG, Bad Homburg, Germany) for about 4 hours using dialysate solution (each one liter on concentration solution contains Na 103 mmol, K 2 mmol, Ca 1.75 mmol, Mg 0.5 mmol, Cl 109.5 mmol, and NaCHO_3_ 35 grams). Twenty age and sex matched controls recruited from children attending the general outpatient paediatrics clinic and who had no history of either renal or eye problems were also investigated. Ethical approval for the study was obtained from the institutional research committee and written informed consents were obtained from all parents of children included in the study. Subjects underwent full ophthalmologic examination and the presence of dry eye symptoms was noted. Tear function tests (including Schirmer's test and tear film break-up time [TBUT] test) as well as impression cytology for evaluation of conjunctival squamous metaplasia were performed for all children.

Exclusion criteria included children who use contact lenses, who had previous ocular trauma, who had prior ocular surgery, who had refractive errors, and/or who are taking topical medication. Also children with suspected (either clinically or laboratorially) diabetes mellitus, thyroid dysfunction, metabolic disorders, syndromes with facial abnormalities, or organomegaly or mentally retarded children were excluded from the study.

Careful history taking including the onset and duration of dialysis, the main cause of renal failure, positive family history of similar conditions (family history of child with similar diseases or previous death due to renal diseases), and medications used (as Ca carbonates, vitamin D, folic acid, erythropoietin hormone, iron preparations, and antihypertensive medications), and thorough clinical examination was done for all children including vital data (pulse, temperature, and blood pressure) and anthropometric measurements (body weight in kg, length in cm, and body mass index (BMI)) and careful systematic examination for head and neck, cardiovascular, chest, abdominal, and musculoskeletal systems was done.

All participants underwent a full ophthalmologic examination including best corrected visual acuity (VA) using the Snellen chart, slit lamp biomicroscopic examination, tear function tests including Schirmer's test and TBUT, fundoscopy, intraocular pressure, and the autorefractometer (Nidek-AR-1a, NidekCo., Ltd., Japan) being used to detect refractive errors in them.

The TBUT test was performed by instilling one drop of fluorescein solution into the conjunctival sac without using topical anaesthetic. Tear film was observed with a cobalt blue filter under wide lighting using. The interval between last blink and the appearance of a corneal dry spot in the stained tear film was measured. This procedure was repeated three times and the average value was used. Less than 10 s was considered abnormal.

Basal Schirmer's test was performed after one drop of topical anaesthetic instillation using benoxinate hydrochloride. Standardised Whatman filter paper secured the lateral cantus away from the cornea and was left in place for 5 min. The amount of wetting was measured in millimetres. Measurements less than 10 mm were considered insufficient secretion.

The status of conjunctival epithelium was evaluated by impression cytology and the expression of CK5/6 molecule was investigated by immunocytochemistry to evaluate squamous differentiation [10, 11]. Samples of both the right and left eyes of each subject were obtained from the temporal bulbar conjunctiva using a cotton probe. The samples were quickly smeared on prelabelled coated slides, before fixation for 30 minutes at 95% ethyl alcohol, and stained with the standard H&E stain. For immunocytochemistry, the smears were incubated in 0.5% hydrogen peroxide for 10 minutes to block endogenous peroxidase activity. Antigen retrieval was done by boiling the smears in 10 mM citrate buffer pH 6.0 for 10 minutes using a microwave at high power followed by cooling down at room temperature for 30 minutes. The smears were then washed twice in PBS before incubation with mouse monoclonal anti-CK5/6 (Dako, Cat #M 7237) at a dilution of 1/100 antibody for 30 minutes at room temperature. After washing twice in PBS, the smears were incubated with biotinylated goat anti-polyvalent secondary antibody for 10 minutes at room temperature. The slides were washed twice in PBS, incubated with streptavidin peroxidase for 10 min at room temperature, washed twice in PBS, and exposed to a freshly prepared 3,30-diaminobenzidine tetrahydrochloride solution (DAB) for 10 minutes at room temperature. Finally, the slides were counterstained with hematoxylin, washed in running water, dehydrated in graded alcohol, and mounted as usual. Sections of normal skin were used as positive control for the immunohistochemistry process.

Five mL of venous blood was aspirated under complete aseptic conditions for measurement of complete blood count (CBC) using a Celtic autocounter after calibration; urea, creatinine, calcium, phosphorus and potassium (by fully automated clinical chemistry autoanalyzer system Konelab 20i) liver function tests (using Integra 400 auto analyser), and parathyroid hormone (PTH) were measured by the Vitek Immuno Diagnostic Assay System (VIDAS-BioMerieux, France).

### 2.1. Statistical Analysis

Data were analyzed using STATA intercooled version 12.1. Quantitative data were represented as mean, standard deviation, median, and range. Data were analyzed using Student's *t*-test to compare means of two. When the data were not normally distributed, the Mann-Whitney test was used to compare two groups. Qualitative data were presented as number and percentage and compared using the Chi square test. Correlation between different variable was done by using Spearman's rank correlation test. *P* value was considered significant if it was less than 0.05.

## 3. Results

In this study group, the mean age (SD) was 10.8 (2.82) years, with a range of 6–16 years, while the mean (SD) duration of ESRF disease was 3.73 (1.89) years. The mean (SD) duration of dialysis was 2.5 (1.88) years. Mean (SD) weight was 24.73 (7.48) kg, mean (SD) length was 127.83 (12.04) cm, and mean (SD) BMI was 14.85 (2.23) kg/m^2^. There were no statistically significant differences between the study group and the control group with regard to the best corrected visual acuity using the Snellen chart, slit lamp examination, fundus examinations, intraocular pressure, or any refractive errors. In the study group, a positive family history of a similar condition, the presence of systemic diseases, and cases positive to hepatitis C were 36.67%, 60%, and 26.67%, respectively, as shown in Table 1.

In Table 2, some laboratory finding and blood pressure values of the study group were shown: the mean (SD) serum creatinine level was 5.54 (1.38) mg/dL, the mean (SD) serum urea level was 127.6 (14.30) mg/dL, the mean (SD) total serum Ca level was 8.31 (0.80) mg/dL, the mean (SD) serum phosphate level was 3.19 (1.03) mg/dL, the mean (SD) PTH was 733.17 (417.80) pg/mL, the mean (SD) total protein was 7.29 (0.64) g/dL, the mean (SD) albumin was 3.87 (0.30) g/dL, Hb level was 9.89 (1.74) g/dL, the mean (SD) platelet count was 196.37 (52.16) thousand/dL, the mean (SD) WBC count was 6.46 (1.46) thousand/dL, the mean (SD) serum Na level was 136.03 (3.37) mg/dL, the mean (SD) serum K level was 5.49 (0.79) mg/dL, the mean (SD) systolic blood pressure was 125.5 (18.63) mmHg, and the mean (SD) diastolic blood pressure was 80.83 (13.96) mmHg.

**Figure 1:**
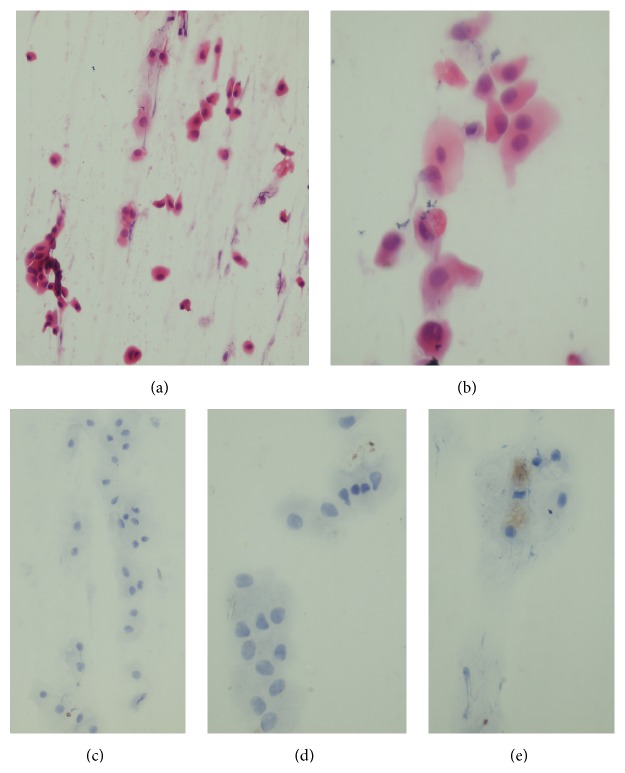
Impression cytology yielded a cellular material of conjunctival columnar cells (a and b). The cells are negative for CK5/6 expression (c and d) apart from weak positive expression in few cells in one case (e). H&E stain (a and b) and CK5/6 immune stains (c, d, and e). Magnification is ×200 (a and c) and ×400 (b, d, and e).

Regarding cytological examination of the conjunctival smears, the imprint smears yielded a generally cellular material (Figure 1(a)) and most of the encountered cells are of columnar morphology (Figure 1(b)). Nearly all evaluated smears showed complete negative immunoreaction of the cells to anti-CK5/6 antibody (Figures 1(c) and 1(d)). Very few cells showed weak cytoplasmic expression of CK5/6 molecule in one case (Figure 1(e)).

In Table 3, there are no significant differences between ESRF children in dialysis and control group regarding the age and gender. However, there were statically significant differences between two groups with regard to the presence of dry eye symptoms. Dry eye symptoms such as itching, redness, burning, foreign body sensation, photophobia, and blurred vision were detected in 26% of children with ESRF on dialysis, compared with none of the controls (*P* = 0.05). There were also significant differences in the study group compared to the control group with regard to the Schirmer test (right eye) (*t* 24.63, *P* = 0.01), the Schirmer test (left eye) (*t* 24.7, *P* = 0.02), TBUT test (right eye) (*t* 11.9, *P* = 0.002), and TBUT test (left eye) (*t* 11.4, *P* = 0.0004) as shown in Table 3.

In Table 4, within the study group, a negative correlation was found between TBUT and Schirmer's test results and the duration of ESRF diseases (*R* = 0.45, *P* = 0.01, and *R* = 0.46, *P* = 0.01, resp.). Also, a negative correlation was found between TBUT and Schirmer's test results and the duration of dialysis (*R* = 0.39, *P* = 0.03, and *R* = 0.45, *P* = 0.01, resp.). No relation was detected between the TBUT and Schirmer's test results and the serum creatinine, urea, Ca, phosphate, PTH, total protein, albumin, Na, K, Hb level, platelet counts, WBCs count, systolic blood pressure, and diastolic blood pressure levels in the study group.

## 4. Discussion

In this prospective, cross-sectional study, thirty children with ESRF on dialysis and 20 age and sex matched controls at the dialysis unit of paediatric department, Sohag University, Egypt, were included in the study. The basal tear secretion and tear film stability (tested by TBUT and Schirmer tests) were lower, and the dry eye symptoms were more common among children with ESRF. The duration of ESRF and dialysis statuses seems to be related with the disturbances in tear secretion and tear film stability.

Presence of dry eye manifestations such as redness, itching, and irritation was found in about 26% of the study and none were seen in the control group; this agrees with Akinci et al. [4] study who studied the ocular abnormalities in 19 patients with ESRF and found dry eye symptoms in 15.8% of children with ESRF. However, the small number of that study was an obvious disadvantage. Furthermore, in this study about 26.6% of the ESRF children were affected with hepatitis C infection which is described in adults as a cause of many extrahepatic systemic manifestations including thyroid disease (Hashimoto's thyroiditis, Graves' disease, and thyroid cancer), cardiovascular disease (atherosclerosis, carotid artery disease, and coronary artery disease), renal disease (membranoproliferative glomerulonephritis and glomerulosclerosis), and eye disease (Mooren's ulcers and sicca syndrome). These manifestations are collectively called C hepatitis-associated systemic manifestations (CHASMs) [12]. However, we did not find any correlation between the presence of positive hepatitis C infection in children with ESRF on dialysis and any abnormal tear functions tests.

Moreover, Behbehani et al. [5] reviewed the records of ophthalmic assessments in children with ESRF before renal transplantation and detected abnormalities in 46 (43%) patients out of 107 patient charts; the abnormalities had not been detected previously in 14 (13%), while vision-threatening eye disorders were found in 6 (6%) of the patients. Behbehani et al. concluded that children with ESRF had a high prevalence of ocular abnormalities, but most of the abnormalities did not affect visual function; this agrees well with our results, as no case from our study group had abnormalities affecting visual function.

Nevertheless, Al Mosawi [13] retrospectively assessed the records of 80 children with ESRF for a long period of about 14 years with or without dialysis. He found that corneal cysteine crystals had formed in 6 patients (7.5%), congenital cataract and glaucoma being seen in 3 patients (3.75%). This is not the case in our study, as, in the Al Mosawi study, he included hereditary disorders and genetic syndromes accounting for 28.75% of children with ESRF, with nephropathic cystinosis as the most common hereditary disorder causing ESRF, but, in our study, we excluded all syndromes with facial abnormalities or organomegaly or mentally retarded children. Moreover, Krause et al. [6] found retrospectively some ocular complications in postrenal transplantation children. They found that cataract was the most common finding (8.4% patients) followed by swollen disk (5.7% patients), hypertensive retinopathy (5.7% patients), and increased intraocular pressure (3% patients). There was a complete lack of any of our findings in Krause et al. [6] report that might hint to the complete reversibility of the corneal and tear abnormalities with renal transplantation.

Schirmer's test values were found to be significantly lower in ESRF than in control subjects. Undoubtedly, Schirmer's test is rather a rough screening test for the detection of tear hyposecretion than a technique for the precise measurement of tear production. The sensitivity of Schirmer's test could be shown to be as low as 10–30% [14, 15]. On the other hand, when performed in a standardized procedure, the finding of a statistically significant difference between Schirmer's test values of two groups may provide valuable information on the amount of stimulated tear secretion. Thus, the present data suggest that, on average, the amount of provoked reflex tearing is lower in ESRF children than in normal children.

ESRF duration and dialysis duration had a statistically significant impact on ocular manifestation and tear function test in our study, which agrees with the results of Akinci et al. [4]. These results may be explained by elevated calcium-phosphate product secondary to hyperparathyroidism as the mean of parathyroid hormone level was increased in those children up to 733.17 pg/mL (normal level up to one hundred pg/mL). Secondary hyperparathyroidism leads to calciphylaxis with corneal deposition of calcium-phosphate crystals being one of the consequences of this state and it might explain part of the findings in this study [16]. Other factors mentioned in this study such as serum creatinine level, serum electrolytes, and blood pressure had no effect on TBUT and Schirmer's test.

Impression cytology of the conjunctival surface showed distinctly more frequent and more pronounced signs of squamous metaplasia of the conjunctival epithelium in ESRF patient in adult and old age [7–9]. To our knowledge there were no previous investigations for the status of the conjunctival epithelium in children with ESRF especially in Egypt. In this study, almost all cases showed no evidence of squamous metaplasia based on the ordinary cytological evaluation or more accurately by assessment of CK5/6 molecule that is specific for squamous epithelium [10, 11]. There are only very few countable cells in one case: a 15-year-old girl on dialysis since the age of 8 showed early squamous metaplastic changes based on weak expression of CK5/6 (Figure 1(e)). This implies that the development of conjunctival squamous metaplasia which is a major morphological cellular change occurs after long-term dialysis. On support for this contention, Demir and his colleagues [2] reported that severity of conjunctival squamous metaplasia is related to the duration of ESRF. They showed that 44% of adult patients with ESRF for more than 10 years showed conjunctival squamous metaplasia compared to 22% of patients with CRF of less than 10-year duration. Moreover, in another study of adult patients with ESRF by Bakaris et al. [9], they found no correlation between impression cytology and calcium deposit grades but the presence of conjunctival inflammation correlated with the existence of extensive squamous metaplasia. This implies that the development of conjunctival squamous metaplasia in ESRF occurs after a long-term dialysis.

Patients on chronic dialysis and especially children are predisposed to malnutrition and indeed this seems to be the case for this cohort whose mean weight is 24.7 kg at a mean age of 11 years as well as BMI 14.85 kg/m^2^ (below 3rd percentile for both girls and boys at this age) which reflect the state of vitamins and minerals deficiency which play a golden role in all organ functions including the eye and lacrimal glands [17].

In this study the mean values for systolic blood pressure (125 mmHg) which were between 90th and 97th percentile and diastolic blood pressures (80 mmHg) which were between 85th and 97th percentile according to WHO charts for blood pressure [18] were higher than controls and this was expected secondary to renal ischemia resulting in activation of renin-angiotensin system and secondary elevation of blood pressure [19]. Fortunately, in children of this study the mean intraocular pressure was normal corresponding to their age group. There was no affection of higher blood pressures on the retina or visual acuity. Furthermore, there were no significant correlations between the blood pressure and the tear functions excluding the blood pressure as a cause of tear functions disorders in ESRF patients in the present study. With regard to the drugs received by our patients in the form of supplementary vitamin D, calcium carbonate, iron, folic acid, and erythropoietin hormone and methyldopa, all of them do not affect the tear functions [20, 21].

## 5. Conclusions

The basal tear secretion and tear film stability were lower while the dry eye symptoms such as itching and redness were more common among children with ESRF. The duration of ESRF and dialysis duration seem to be related to the disturbances in tear secretion and tear film stability. However, squamous metaplasia of the conjunctival epithelium is very rare. Routine ophthalmological examination for children with ESRF on dialysis is suggested for early detection of eye abnormalities.

## Figures and Tables

**Table 1:** Patients' characteristic of study group.

Patients' characteristic	Summary statistics
Age	
Mean (SD)	10.8 (2.82)
Median (range)	11 (6–16)
Gender	
Females	16 (53.33%)
Males	14 (46.67%)
Weight/kg	
Mean (SD)	24.73 (7.48)
Median (range)	23 (13–49)
Length/cm	
Mean (SD)	127.83 (12.04)
Median (range)	125.5 (108–155)
BMI kg/m^2^	
Mean (SD)	14.85 (2.23)
Median (range)	14.89 (9.03–20.40)
Duration of disease/year	
Mean (SD)	3.73 (1.89)
Duration of dialysis/year	
Mean (SD)	2.5 (1.88)
Family history	
Negative	19 (63.33%)
Positive	11 (36.67%)
Systemic disease	
No	12 (24.00%)
Yes	18 (60.00%)
Hepatitis C virus	
Negative	22 (73.33%)
Positive	8 (26.67%)

**Table 2:** Laboratory finding and blood pressure of study group.

Patients' characteristic of study group	Summary statistics
Creatinine (mg/dL)	
Mean (SD)	5.54 (1.38)
Urea (mg/dL)	
Mean (SD)	127.6 (14.30)
Ca (mg/dL)	
Mean (SD)	8.31 (0.80)
Phosphate (mg/dL)	
Mean (SD)	3.19 (1.03)
Parathyroid hormone (pg/mL)	
Mean (SD)	733.17 (417.80)
Total protein (g/dL)	
Mean (SD)	7.29 (0.64)
Albumin (g/dL)	
Mean (SD)	3.87 (0.30)
HB (g/dL)	
Mean (SD)	9.89 (1.74)
Platelets (thousand/dL)	
Mean (SD)	196.37 (52.16)
WBCs (thousand/dL)	
Mean (SD)	6.46 (1.46)
Na (mg/dL)	
Mean (SD)	136.03 (3.37)
K (mg/dL)	
Mean (SD)	5.49 (0.79)
Systolic blood pressure (mmHg)	
Mean (SD)	125.5 (18.63)
Median (range)	125 (90–150)
Diastolic blood pressure (mmHg)	
Mean (SD)	80.83 (13.96)
Median (range)	80 (50–100)

**Table 3:** Comparison between study and control groups.

Item	Study group, 30 children	Control group, 20 children	*P* value
Age in years			
Mean (SD)	10.8 (2.82)	9.50 (2.48)	0.10
Median (range)	11 (6–16)	10.55 (6–16)	
Gender			
Females	16 (53.33%)	8 (40%)	0.31
Males	14 (46.67%)	12 (60%)	
Presence of dry eye manifestations			
No	22 (73.00%)	20 (100%)	0.05
Yes	8 (26.00%)	0	
Schirmer's test (right eye) seconds			
Mean (SD)	24.63 (7.03)	29.55 (5.12)	0.01
Median (range)	25 (7–35)	30 (20–35)	
Schirmer's test (left eye) seconds			
Mean (SD)	24.7 (8.11)	29.7 (4.84)	0.02
Median (range)	27 (5–35)	30 (20–35)	
Tear film break-up time (TBUT) test (right eye) seconds			
Mean (SD)	11.9 (2.55)	13.85 (1.04)	0.002
Median (range)	12 (5–15)	14 (12–15)	
Tear film break-up time (TBUT) test (left eye) seconds			
Mean (SD)	11.4 (2.88)	14.0 (1.17)	0.0004
Median (range)	12 (4–15)	14.5 (12–15)	
Squamous metaplasia of the conjunctival epithelium (right and left eye)			
Not detected	29 (96.7%)	20 (100%)	0.409
Detected	1 (3.3%)	0	

**Table 4:** Correlation between TBUT and Schirmer's test results and ESRF-related variables.

Variables	TBUT		Schirmer's test	
	*R*	*P*	*R*	*P*
Duration of the disease	−0.45	0.01	−0.46	0.01
Duration of dialysis	−0.39	0.03	−0.45	0.01
Creatinine	0.03	0.88	−0.03	0.89
Urea	−0.06	0.72	−0.05	0.77
Ca	−0.26	0.15	−0.29	0.13
Phosphate	0.15	0.41	0.10	0.60
Parathyroid hormone (PTH)	0.20	0.28	0.13	0.46
Total protein	0.33	0.55	0.13	0.67
Albumin	0.53	0.32	0.27	0.16
Na	0.20	0.29	0.14	0.45
K	0.09	0.60	0.04	0.83
HB	0.13	0.50	0.03	0.87
Platelets	0.23	0.12	0.29	0.13
WBCs	0.29	0.12	0.30	0.11
Systolic blood pressure	−0.12	0.34	−0.21	0.27
Diastolic blood pressure	−0.12	0.54	−0.18	0.35
